# JointLIME: An interpretation method for machine learning survival models with endogenous time‐varying covariates in credit scoring

**DOI:** 10.1111/risa.17679

**Published:** 2024-11-20

**Authors:** Yujia Chen, Raffaella Calabrese, Belen Martin‐Barragan

**Affiliations:** ^1^ Business School University of Edinburgh Edinburgh UK; ^2^ European University Institute Via dei Roccettini, Fiesole Tuscany Italy

**Keywords:** explainable AI, joint model, machine learning, survival analysis

## Abstract

In this work, we introduce JointLIME, a novel interpretation method for explaining black‐box survival (BBS) models with endogenous time‐varying covariates (TVCs). Existing interpretation methods, like SurvLIME, are limited to BBS models only with time‐invariant covariates. To fill this gap, JointLIME leverages the Local Interpretable Model‐agnostic Explanations (LIME) framework to apply the joint model to approximate the survival functions predicted by the BBS model in a local area around a new individual. To achieve this, JointLIME minimizes the distances between survival functions predicted by the black‐box survival model and those derived from the joint model. The outputs of this minimization problem are the coefficient values of each covariate in the joint model, serving as explanations to quantify their impact on survival predictions. JointLIME uniquely incorporates endogenous TVCs using a spline‐based model coupled with the Monte Carlo method for precise estimations within any specified prediction period. These estimations are then integrated to formulate the joint model in the optimization problem. We illustrate the explanation results of JointLIME using a US mortgage data set and compare them with those of SurvLIME.

## 1 INTRODUCTION

Survival methods are widely used and well‐developed in credit risk modeling (Bellotti & Crook, 2009; Calabrese & Crook, 2020; Dirick et al., 2019; Medina‐Olivares et al., 2022; Stepanova & Thomas, 2002; Xie et al., 2019). Compared to static classification approaches, which can only model the probability of default (PD) in a predefined time window, survival methods can model when and who are likely to default (Thomas et al., 2017). The most prevalent survival model is the Cox model (Allison, 1982; Cox, 1972), which considers a proportional hazard model for continuous time and a proportional odds model for discrete time. The Cox model initially only considers time‐invariant covariates, but it can also be extended to include time‐varying covariates (TVCs). In the credit scoring context, it has been shown that the inclusion of TVCs such as macroeconomic and behavioral variables can significantly improve the model fit and the accuracy of predictions (Bellotti & Crook, 2009, 2013; Djeundje & Crook, 2018; Leow & Crook, 2016).

TVCs are distinct and can be categorized into two types: endogenous and exogenous. The criterion for this distinction is whether the future paths of the variables are influenced by the individual's survival status (default). Variables that are not influenced are exogenous TVCs, such as macroeconomic variables, including GDP and the unemployment rate of a region, among others. On the other hand, variables that are influenced are endogenous TVCs. Therefore, endogenous TVCs are key variables in survival analysis since they carry direct information on the time to the default. Examples of endogenous TVCs in the credit scoring context could be the outstanding balance and arrears in instalments, which are behavioral variables (Hu & Zhou, 2019; Medina‐Olivares et al., 2023, 2022).

However, the Cox model itself can only handle exogenous TVCs. Previous research has indicated that directly including endogenous TVCs in the Cox model (i.e., assumed as exogenous TVCs), usually known as the extended Cox model in the literature, can lead to estimation biases (Dafni & Tsiatis, 1998; Rizopoulos, 2012; Tsiatis et al., 1995). In the credit scoring literature, where models are primarily developed for predictive purposes, measuring bias is often considered less critical. Thus, the distinction between exogenous and endogenous TVCs is commonly overlooked. When including endogenous TVCs in a survival model for credit scoring, the standard method is to make predictions by lagging their values, thereby relating past information to future survival status (Bellotti & Crook, 2013, 2014; Djeundje & Crook, 2018; Leow & Crook, 2016). However, this method has some limitations. First, the lag period on the behavioral variables adversely affects the model estimate due to the absence of these variables during the initial period of survival model estimation, corresponding to the length of the designated lag period. Second, using lagged values in estimation fails to synchronize observations between TVCs and survival paths, which could indeed affect predictive performance, especially when TVCs change significantly over a period shorter than the lag period. In addition, predefining the lag period limits the analysis to a specific time window, which contradicts the flexibility inherent in survival approaches that do not rely on predefined time windows. Finally, if the endogeneity between the TVCs and the survival process is significant, lagging the TVCs does not guarantee the elimination of potential bias.

Therefore, to address the problem of endogeneity, joint models are proposed and can be fitted using either the two‐stage approaches (Albert & Shih, 2010b; Thi et al., 2018; Ye et al., 2008) or the joint likelihood formulation (Albert & Shih, 2010a; Barrett et al., 2015; Medina‐Olivares et al., 2023). Joint models can control measurement errors in observed endogenous TVCs and avoid estimation bias by modeling both longitudinal and survival data and considering a mutual evolution of both processes. Moreover, based on the capability to estimate the random effects of endogenous TVCs for newly collected data, joint models can produce dynamic predictions that do not rely on lagging the TVCs. Hu and Zhou (2019) and Medina‐Olivares et al. (2023, 2022) applied joint models in credit risk prediction and compared them with the Cox model that directly includes observed endogenous TVCs. They found that joint models can yield better predictive performance. Note that “endogenous TVC(s)” are also known as “longitudinal data/outcomes” in the literature on joint models. Here to unify the jargons, we use “endogenous TVC(s)” in this paper.

In addition to the statistical models mentioned above, machine learning (ML) algorithms have been adapted for use in survival analysis and have shown superior predictive performance and computational efficiency in handling complex and large data sets compared to traditional statistical models. The commonly used ML models in survival analysis can be categorized into two main types: models based on ensemble trees (Bai et al., 2022; Frydman & Matuszyk, 2022; Ishwaran et al., 2008; Moradian et al., 2022) and models based on deep neural networks (Blumenstock et al., 2022; Katzman et al., 2018; Lee et al., 2018). Due to the importance of endogenous TVCs, many researchers (Bou‐Hamad et al., 2011; Lee et al., 2019; Lim et al., 2019; Lin & Luo, 2022; Nagpal et al., 2021; Yao et al., 2022; Zhang et al., 2020) developed ML survival models that effectively incorporate endogenous TVCs, leveraging the flexibility of ML methods and their capacity to process arbitrary sequences of inputs (such as with recurrent neural network, or RNN). However, ML survival models work as black‐box models since they are too complex and it is difficult to directly explain and validate their predictions. This leads to the limited application of these approaches in many important areas. For example, in the credit scoring domain, there are regulations such as the Equal Credit Opportunity Act (ECOA) in the United States (Consumer Financial Protection Bureau, 2022) and the General Data Protection Regulation (GDPR) in the EU (Voigt & von dem Bussche, 2017) enacted to emphasize the need for modeling transparency and interpretability in the lending sector.

The lack of interpretability in the black‐box survival (BBS) models has motivated the development of several methods to explain the black‐box predictions and understand the key factors that affect the model output. However, to the best of our knowledge, existing methods for explaining BBS models, such as different versions of SurvLIME (Kovalev et al., 2020; Kovalev & Utkin, 2020; Utkin et al., 2022), only consider time‐invariant covariates. They are not appropriate for handling TVCs, especially endogenous TVCs, since they cannot model and forecast the endogenous TVCs needed to make explanations in the prediction period. Given the importance of endogenous TVCs and the popularity of BBS models that consider such variables, we aim to propose a new interpretation method called JointLIME that fills this gap.

To summarize, this paper contributes to the existing literature by proposing JointLIME, the first method for explaining BBS models with endogenous TVCs. JointLIME differs from previous methods in the following aspects:

- It incorporates the joint model in the explanation framework to better handle the endogenous TVCs instead of the Cox model included in SurvLIME and its variants.
- Inspired by SurvLIME, but different from LIME, in a local area around a new individual, it constructs an optimization problem to minimize the distances between the survival functions predicted by the BBS model and those formulated by the joint model.
- By leveraging the joint model, once the optimization problem is solved, it can provide a unique importance value (i.e., absolute coefficient value in the joint model) for each covariate included in the prediction, regardless of whether it is time‐invariant or time‐varying. In contrast, a direct application of SurvLIME would give an importance value for each time point for the endogenous TVC, which would be unrealistic, unreliable, and difficult to interpret.
- It employs a spline‐based approach to model the endogenous TVC in the joint model instead of the commonly used linear mixed‐effects model to obtain more reliable results in fitting and predicting the future values of endogenous TVC.
- For a new individual, it uses a Monte Carlo approach in the joint model to obtain the estimates of the true and unobserved values of the endogenous TVC (i.e., endogenous TVC without measurement errors) within any specified prediction period. In contrast, SurvLIME lacks an endogenous TVC modeling phase, thereby failing to create complete estimates of endogenous TVC for a new individual and failing to provide effective explanations.
- It treats survival time as discrete, rather than the continuous format assumed by any other interpretation methods. This aligns with the intrinsic nature of credit risk modeling, where account records are typically observed monthly, thereby rendering events fundamentally discrete.

We present an illustration of the explanation results obtained by JointLIME in the context of credit scoring, using a US mortgage data set and compared with those of SurvLIME.

The rest of the paper is structured as follows. Section 2 briefly reviews the proposed interpretation methods and we focus on the methods developed to explain the black‐box models in survival analysis. Section 3 introduces the basic concepts of survival analysis, followed by a brief introduction of Dynamic‐DeepHit, the BBS model used in this paper for generating predictions in Section 4. Then, the details of the joint model used in JointLIME are presented in Section 5. Section 6 thoroughly describes the explanation framework of JointLIME. Section 7 presents an application of JointLIME to the US mortgage data set and offers a comparative analysis of explanation results between JointLIME and SurvLIME. Conclusion and discussion of future research can be found in Section 8.

## 2 LITERATURE REVIEW

With the increasing emphasis on the interpretability of ML models, a great number of interpretation methods have been proposed and utilized in different applications in recent years (Barbaglia et al., 2021; Molnar, 2021; Yao et al., 2023). Some methods, such as Partial Dependence Plots (Friedman, 2001) and Accumulated Local Effects Plots (Apley & Zhu, 2020), can be seen as global interpretation methods since they help in understanding how different variables in a data set influence the predictions of an ML model on an average level.

In addition to global methods, a second approach is a local explanation that focuses on individual predictions. For example, LIME proposed by Ribeiro et al. (2016) belongs to this framework. It aims to explain the ML model prediction of a specific individual by approximating the predictions made by the black‐box model with an interpretable model (e.g., Ridge regression) at a local area around the individual. Many modifications based on LIME, such as Anchors (Ribeiro & Guestrin, 2018), ALIME (Shankaranarayana & Runje, 2019), DLIME (Zafar & Khan, 2019), and so forth, have been proposed to achieve better explanation stability and fidelity. In addition to LIME, another popular local interpretation method is SHapley Additive exPlanations (SHAP) (Lundberg & Lee, 2017; Lundberg et al., 2020), which computes the contribution of each variable to the prediction for an individual based on the Shapley values. There are also counterfactual explanations (Dandl et al., 2020; Wachter et al., 2018) based on the smallest change to the variable values of an individual needed to modify the predefined outcome.

Many researchers have also provided comprehensive and critical reviews of interpretation methods (Barredo Arrieta et al., 2020; De Bock et al., 2023; Guidotti et al., 2018; Molnar, 2021).

All of the interpretation methods mentioned above have been employed in the credit scoring domain (Barbaglia et al., 2021; Bussmann et al., 2021; Bücker et al., 2021; Chen et al., 2024; Dandl et al., 2020). It should be noted that these interpretation methods can only explain point‐valued predictions produced by black‐box models, for example, predictions of default probability in a predefined time window in credit scoring. However, as mentioned in Section 1, survival analysis models generate predictions of hazard or survival functions, which provide a representation of the predicted default as a function that captures variation over time. Therefore, interpretation methods capable of explaining function‐valued predictions are needed to understand BBS models.

Kovalev et al. (2020) proposed SurvLIME, an extension of LIME, to approximate the cumulative hazard function (CHF) predicted by a BBS model using the CHF produced by the Cox proportional hazard model for the same individual. This allows the coefficients of variables in the Cox model to be regarded as the variable importance values, thereby serving as an explanation for the predictions of the BBS model. Specifically, SurvLIME works as follows: first, a neighborhood data set is generated around the target individual. The coefficients of variables in the Cox model are then fitted by minimizing the distances between two CHFs derived, respectively, from the BBS model and the Cox model for each individual in the neighborhood data set. A variant of SurvLIME, called SurvLIME‐Inf, was proposed by Utkin et al. (2020) uses $L_{\infty }$‐norm for defining distances between the CHFs instead of $L_{2}$‐norm in SurvLIME. $L_{\infty }$‐norm helps to achieve a simpler linear programming problem for determining important variables and a better explanation performance when the training set is small. Kovalev and Utkin (2020) further modified SurvLIME‐Inf to incorporate the Kolmogorov–Smirnov bounds to approximate the CHFs of the BBS model, achieving robustness explanations. SurvNAM (Utkin et al., 2022) uses generalized additive models (GAMs) to model the relationship of covariates instead of the simple linear expression used in SurvLIME. The GAM is implemented using the neural additive model (NAM), trained with a specific loss function that accounts for disparities between the CHFs derived from the BBS and the Cox models. While explanations in SurvLIME and its variations are depicted by single values gauging variable importance, SurvNAM presents explanations in the form of variable shape functions, indirectly illustrating the variable impact. All these methods are designed for time‐invariant covariates and the TVCs case has not been explored.

Counterfactual explanations and SHAP extensions have also been proposed in the literature to explain BBS models. Kovalev et al. (2021) presented a method to find counterfactual explanations for predictions of BBS models, which is based on analysis of survival functions. This method measures the difference between the original individual of interest and the counterfactual. It calculates this difference by comparing the mean times to the event obtained from the survival functions of both the original individual and the counterfactual. Moreover, Krzyziński et al. (2023) proposed SurvSHAP(t) that preserves the desired SHAP properties (local accuracy, missingness, and consistency stated in Lundberg and Lee, 2017) and extended to consider the time‐dependent nature of the explanation in the survival analysis. Specifically, SurvSHAP(t) assigns an importance value to each variable at each time point, thereby describing a time‐dependent influence of variables on the predicted survival function produced by the BBS model. Again, the aforementioned interpretation methods are limited to BBS models that include only time‐invariant covariates.

As highlighted in Section 1, endogenous TVCs are crucial for enhancing prediction accuracy and they are often used in BBS models. This paper contributes to filling this gap by presenting JointLIME, a new method that can effectively handle endogenous TVCs through a joint modeling framework. JointLIME provides explanations for predictions based on all time‐invariant covariates and TVCs used in the BBS model.

## 3 BASIC CONCEPTS OF SURVIVAL ANALYSIS

In this paper, we consider $T\in Z_{+}$ as a discrete random variable which represents the number of months since a loan originated and T takes values j ($j=1,\text{…},J_{i}$) for each loan i (i=1,…,N). We further denote the survival data as a sequence of binary indicators $\left\{x_{ij}\right\}$ such that $x_{ij}=1$ if the event happens at time j and 0 otherwise. Moreover, for each individual i, we use $u_{i}$ to represent the time‐invariant covariates and $y_{ij}$ to represent the endogenous TVCs. Note that, in general, endogenous TVCs may have been measured at time points that are different from the survival information. Therefore, the number of values of endogenous TVCs can differ from the survival times. However, we only consider here the endogenous TVCs measured at regular time intervals (i.e., monthly) and no missing observations before $J_{i}$, which is a typical setting in credit scoring (Medina‐Olivares et al., 2022). Therefore, we can use $y_{ij}$ to indicate every time point the endogenous TVCs are measured before the event or the right‐censoring happens. Then, the data set can be denoted as $D_{N}={\left\{\left(Y_{i},X_{i},u_{i}\right)\right\}}_{i=1}^{N}$ comprising the survival data $X_{i}=\left\{x_{ij}:j=1,\text{…},J_{i}\right\}$, the endogenous TVCs $Y_{i}=\left\{y_{ij}:j=1,\text{…},J_{i}\right\}$, and the time‐invariant covariates $u_{i}$.

In the survival analysis, hazard and survival functions are key concepts for modeling the distribution of time to the event of interest. The discrete hazard function (DHF) denoted by $h_{ij}$ calculates the conditional probability of the event happening at time j given that an individual i has survived until j−1:

(1) $$h_{ij}=P\left(x_{ij}=1|x_{ij-1}=0,Y_{i},u_{i}\right).$$

The probability of surviving beyond a particular time t can be obtained as the product of the conditional survival probabilities for all the time points up to j, such that j≤t. Thus, the survival probability representing the probability of nondefault up to time j is given by

(2) $$S_{ij}=\sum_{t=1}^{j}\left(1-h_{it}\right).$$

## 4 BBS MODEL: DYNAMIC‐DEEPHIT

In this section, we briefly introduce a widely used BBS model known as Dynamic‐DeepHit (Lee et al., 2019). We apply this model for predictions of new individuals since it considers endogenous TVCs and is built based on a discrete time horizon.

### 4.1 Brief overview

Dynamic‐DeepHit is a deep learning approach that incorporates the available endogenous TVCs comprising various repeated measurements. It issues dynamically updated survival predictions for one or multiple competing risk(s) in a fixed and discrete time horizon and we use s ($s=1,\text{…},T_{max}$)^1^ to represent the time point. Specifically, this BBS model employs an RNN structure with a temporal attention mechanism to encode the information in endogenous TVCs into a context vector. Then, for each competing event, a feed‐forward neural network (FNN) composed of fully connected layers is utilized. It takes the context vector and the last measurement as input and estimates the joint distribution of the first hitting time and the competing event.

Finally, for each individual i in the data set $D_{N}$, every output node represents the probability of having event e at time s, that is, $o_{i,s,e}=\hat{P}\left(T_{i}=s,e=e|Y_{i},u_{i}\right)$. Since we only consider one event (i.e., default) without any competing event, the output node is exactly the estimated PD at time s, that is, $o_{is}=\hat{P}\left(T_{i}=s|Y_{i},u_{i}\right)$. Therefore, the estimated survival probability $S_{is}^{BB}$ for an individual i up to time s can be calculated as follows:

$$S_{is}^{BB}=1-\sum_{t=1}^{s}o_{it}.$$

Based on the trained Dynamic‐DeepHit on the data set $D_{N}$, we can obtain the predicted PD at time s for a new individual k that is not included in $D_{N}$, that is, $o_{ks}=\hat{P}\left(T_{k}=s|Y_{k},u_{k}\right)$. Then, the predicted survival probability $S_{ks}^{BB}$ for the new individual k up to time s can be calculated as

(3) $$S_{ks}^{BB}=1-\sum_{t=1}^{s}o_{kt}.$$

### 4.2 Parameter tuning

Implementation of Dynamic‐DeepHit requires setting several hyperparameters. Here, we use a random search method to find the hyperparameter combination based on the fivefold cross‐validation analysis. We randomly select 50 hyperparameter combinations.^2^ They are obtained by randomly selecting one value from a previously defined set of possible values for each hyperparameter. In this paper, nine hyperparameters are considered for the random search and Table 1 reports the sets of possible values for each hyperparameter (Blumenstock et al., 2022; Lee et al., 2019). Specifically, we first divide the training set into five folds. and each fold will be used as the out‐of‐sample validation set. We then evaluate the discriminative performance^3^ using one of the 50 hyperparameter combinations. This evaluation is based on the model trained on the data from the other four folds. Finally, the combination with the best discriminative performance (highest average time‐dependent concordance index over five folds) will be chosen as the final hyperparameter combination and will be used to train the model based on the whole training set.

**TABLE 1:** Hyperparameters tuning sets of Dynamic‐DeepHit.

Hyperparameters	Tuning set
Minibatch size	{32,64,128}
Nonlinearity in RNN	{ReLU,eLU,tanh}
No. of nodes in RNN	{50,100,200,300}
No. of layers in RNN	{1,2,3}
Nonlinearity in temporal attention mechanism	{ReLU,eLU,tanh}
No. of nodes in temporal attention mechanism	{50,100,200,300}
Nonlinearity in FNN	{ReLU,eLU,tanh}
No. of nodes in FNN	{50,100,200,300}
No. of layers in FNN	{1,2,3,5}

Abbreviations: FNN, feed‐forward neural network; RNN, recurrent neural network.

## 5 INTERPRETABLE MODEL: JOINT MODEL

JointLIME utilizes the concept of LIME to fit an interpretable model in the local area around a new individual to explain the predictions. Therefore, to explain the predicted survival function $S_{ks}^{BB}$ produced by the BBS model, we formulate the survival function using the joint model. A standard joint model consists of two submodels—a longitudinal model that fits the endogenous TVC, and a survival model that estimates the event time distribution considering the effect of the endogenous TVC (Rizopoulos, 2012).

### 5.1 Longitudinal submodel—Handling endogenous TVCs

For the longitudinal model, the observed endogenous TVC $y_{ij}$ are assumed to be the true and unobserved value of the endogenous TVCs $m_{ij}$ plus the error terms $\varepsilon_{ij}$, that are assumed to be mutually independent and normally distributed $\varepsilon_{ij}∼N\left(0,\sigma^{2}\right)$:

(4) $$y_{ij}=m_{ij}+\varepsilon_{ij}.$$

We now focus on modeling $m_{ij}$. Instead of the commonly used linear mixed‐effects model in the credit risk modeling (Hu & Zhou, 2019; Medina‐Olivares et al., 2022), we adopt a spline‐based approach (Brown et al., 2005; Rice & Wu, 2001; Wang et al., 2023). We fit the endogenous TVC based on the training data set $D_{N}$. Using a spline‐based approach allows for more flexible and nonlinear shapes for the evolution of the endogenous TVC on an individual level:

(5) $$y_{ij}=\underset{m_{ij}}{\underset{︸}{\sum_{d=1}^{q}\left(\beta_{d}+b_{id}\right)B_{d}\left(j\right)}}+\varepsilon_{ij},\;j=1,\text{…},J_{i},$$

where ${\left\{B_{d}\left(\cdot \right)\right\}}_{d=1}^{q}$ is a q‐dimensional cubic B‐spline basis on $\left[1,T_{max}\right]$ with a fixed knot sequence, $\beta ={\left(\beta_{1},\text{…},\beta_{q}\right)}^{⊤}$ is a vector of fixed effects, and $b_{i}={\left(b_{i1},\text{…},b_{iq}\right)}^{⊤}$ is a vector of random effects for the individual i. $b_{i}^{\prime }s$ are assumed as mutually independent, independent from the error terms $\varepsilon_{ij}$, and coming from a zero‐mean multivariate Gaussian distribution, that is, $b_{i}∼N_{q}\left(0,\Sigma \right)$.

The performance of Equation (5) depends on the number of knots and their locations for the cubic B‐spline basis. In this paper, we place interior knots at the quantiles of $\left[1,T_{max}\right]$, and then determine the number of knots based on the AIC and BIC (Akaike and Bayesian Information criteria) which is widely used for selecting the proper number of knots (Rice & Wu, 2001; Rizopoulos & Ghosh, 2011; Wang et al., 2023).

After obtaining the fitted longitudinal model, for each new individual k that is not included in $D_{N}$, we can obtain the estimates of the true and unobserved value of the endogenous TVC $m_{ks}$ ($s=1,\text{…},T_{max}$) using a Monte Carlo approach (Ribeiro et al., 2016; Rizopoulos, 2012). Specifically, for each Monte Carlo sample, we can obtain an estimate of the random effects $b_{k}^{*}$ for the new individual k from the posterior distribution $\left\{b_{k}|y_{kj},\Phi \right\}$, where $y_{kj}$ denotes the observed endogenous TVC at $j=1,\text{…},J_{k}$ of the new individual k and Φ represents the fitted parameters for Equation (5) using the training set. We then take as the estimate of the random effects $\hat{b_{k}}$ the mean of M estimates $b_{k}^{*\prime }s$ from all M Monte Carlo samples. Given the estimate of the random effects $\hat{b_{k}}$ and the fitted fixed effects $\hat{\beta }$, we can calculate the estimate of $m_{ks}$ for the new individual k at each time point s using $m_{ks}=\sum_{d=1}^{q}\left(\hat{\beta_{d}}+\hat{b_{kd}}\right)B_{d}\left(s\right)$.

### 5.2 Survival submodel

Concerning the survival part in the joint model, following Albert and Shih (2010a, 2010b), Barrett et al. (2015), Medina‐Olivares et al. (2022), and Rizopoulos (2012), we adopt a logit link function to formulate the DHF ($h_{ks}^{JM}$). The survival and longitudinal parts are linked through $m_{ks}$:

(6) $$h_{ks}^{JM}={logit}^{-1}\left(a_{s}^{0}+\gamma^{⊤}u_{k}+\lambda m_{ks}\right),$$

where $a_{s}^{0}$ is the baseline DHF and will be fitted based on the training set $D_{N}$ by using the Nelson–Aalen estimator (Kovalev et al., 2020). γ is the vector of coefficients for the time‐invariant covariates $u_{k}$, and λ is the association coefficient measuring the association between the longitudinal part and the hazard of an event.

Finally, the survival function $S_{ks}^{JM}$ for each individual k up to time s can be formulated by taking account of the DHFs in Equation (6) for all times until s:

(7) $$S_{ks}^{JM}=\prod_{t=1}^{s}\left(1-h_{kt}\right)=\prod_{t=1}^{s}\left(1-{logit}^{-1}\left(a_{t}^{0}+\gamma^{⊤}u_{k}+\lambda m_{kt}\right)\right).$$

γ and λ will be computed by solving an optimization problem to make sure $S_{ks}^{JM}$ is as close as possible to the predicted $S_{ks}^{BB}$ (more details in Section 6).

## 6 A NOVEL INTERPRETATION METHOD: JOINTLIME

In this section, we first introduce the framework of JointLIME in Section 6.1. The data generation process of the JointLIME framework is explored in Section 6.2. The minimization of the distances between the functions is presented in Section 6.3.

### 6.1 JointLIME framework

The interpretation method JointLIME proposed in this paper is an extension of SurvLIME and SurvLIME‐Inf (Kovalev et al., 2020; Utkin et al., 2020). In order to incorporate endogenous TVCs, we propose to use a joint model as introduced in Section 5 instead of a Cox model, as used in SurvLIME. Besides, we adopt a discretization of the timescale for the time‐to‐event outcome, which is preferred in the credit risk analysis as discussed in Section 1.

**FIGURE 1:**
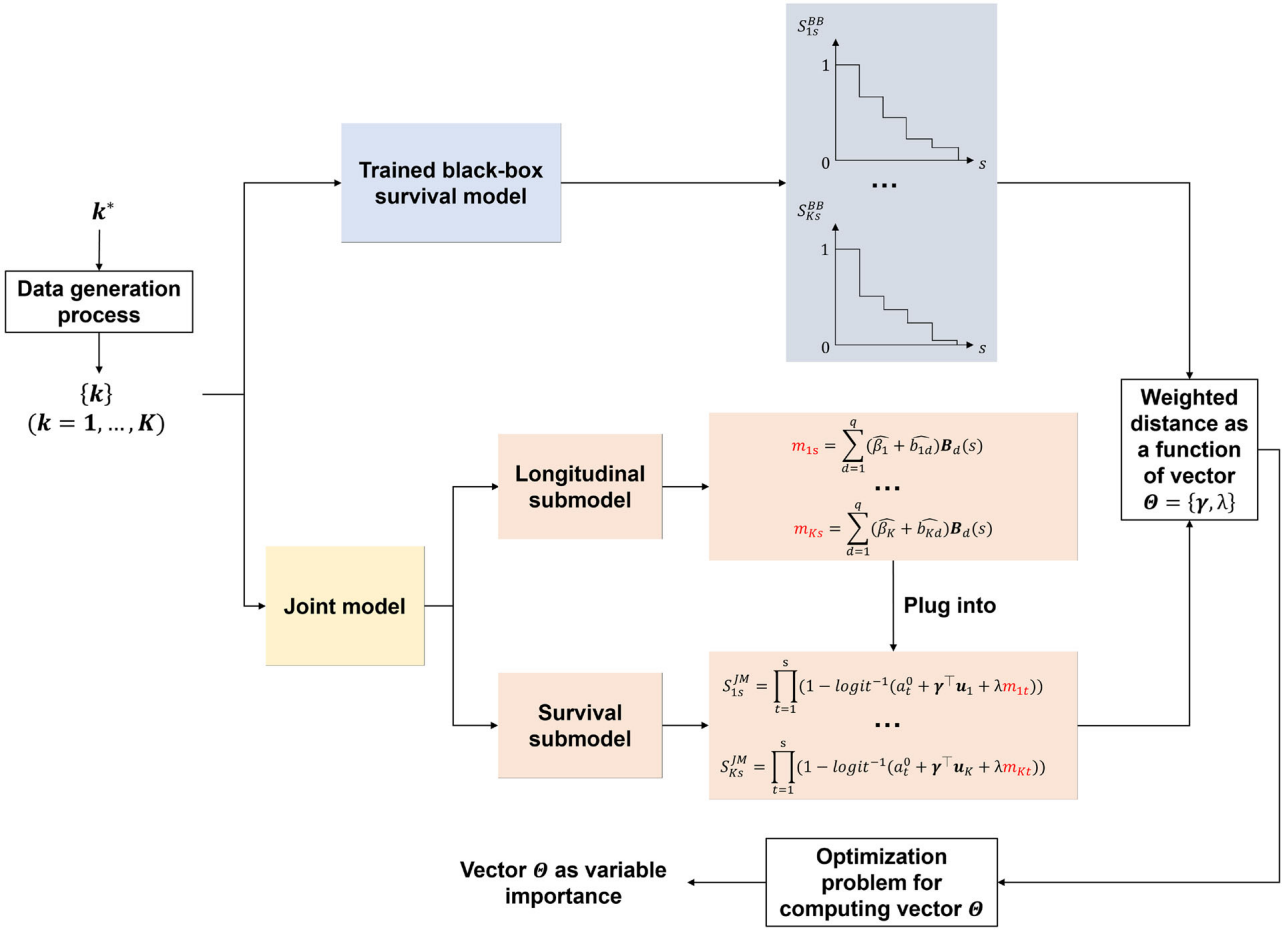
The JointLIME framework.

Figure 1 shows a general scheme of JointLIME. Let us focus on a target individual $k^{*}$. The aim of JointLIME is to approximate the survival function predicted by the BBS model using the survival function produced by the joint model. Therefore, the optimal values of parameters Θ={γ,λ} in the joint model can serve as variable importance values. To achieve this, we first generate a set of new individual {k} (k=1,…,K) in the neighborhood around $k^{*}$.^4^ We would apply the BBS model to each of these individuals, obtaining a prediction of the survival function $S_{ks}^{BB}$. We aim to find the joint model parameters Θ that would result in survival functions $S_{ks}^{JM}\left(|\Theta \right)$ as close as possible to those produced by the black‐box model $S_{ks}^{BB}$. Hence, we compute the parameters Θ that minimizes the sum of the weighted distance of all pairs of survival functions $\left(S_{ks}^{BB},S_{ks}^{JM}\right)$ over the K generated individuals. To ensure the fidelity of the local explanations, there is a weight $w_{k}$ for each pair of survival functions which depends on the distance between each neighborhood individual k and the target individual $k^{*}$. A smaller distance between k and $k^{*}$ means the neighborhood individual is closer to $k^{*}$, thereby producing a larger weight when minimizing the distance between the corresponding pair of survival functions. The above description is an overview of JointLIME, and the details of each step will be discussed in Sections 6.2 and 6.3.

### 6.2 Data generation process

In order to use the joint model to approximate the local behavior of the BBS model, we generate new individuals around the target individual $k^{*}$. To generate a new individual, we assume independence among the covariates.

Therefore, for the time‐invariant covariates, we follow the perturbation method in LIME (Ribeiro et al., 2016) to sample K−1 new values for each variable. Specifically, for each *numerical variable*, we first sample K−1 values from a Gaussian distribution N(0,0.1). Then for these K−1 values, we perform the reverse operation of centering and scaling. This is done based on the value of the corresponding variable for the target individual $k^{*}$ and the standard deviation of that variable in the training data. For each *categorical variable*, we perturb by sampling K−1 values based on the training distribution of the categories of each variable.

For the endogenous TVC, we randomly sample K−1 sequences of endogenous TVC which exist in the training set. Note that it is also possible to consider generating synthetic data for the endogenous TVC. Since it is out of the scope of this paper, we will leave it for future research.

Finally, the generated K−1 values for each variable can form K−1 new individuals. Together with the target individual $k^{*}$, all K individuals can form the neighborhood data set. Note that once we obtain the neighborhood data set, we standardize all the numeric variables to have a zero mean and standard deviation of 1, thereby ensuring the values of the optimal parameters obtained by JointLIME are comparable and can be regarded as variable importance values.

### 6.3 Minimization of distances between functions

ALGORITHM 1 **The algorithm obtaining the parameters Θ for target individual $k^{*}$ using JointLIME**

**Table:** 

**Require**: Training set $D_{N}$, Target individual $k^{*}$, Number of generated individuals K, The BBS model
**Ensure**: Parameters Θ={γ,λ} of important variables of target individual $k^{*}$
**1**: Generate K−1 new individuals and the target individual $k^{*}$ is the K‐th individual
**2**: Compute weight $w_{k}$ using Equation (11) for each individual k, k=1,…,K
**3**: Obtain the prediction of survival function $S_{ks}^{BB}$ for each individual k, k=1,…,K
**4**: Compute the baseline discrete hazard function $a_{s}^{0}$ based on the training set $D_{N}$ using the Nelson‐Aalen estimator
**5**: Fit the longitudinal model using Equation (5) based on the training set $D_{N}$
**6**: Compute the true and unobserved value of the endogenous TVC $m_{ks}$ based on the fitted longitudinal model for each individual k at time s, $s=1,\text{…},T_{max}$
**7**: Formulate the minimization problem with Equation (10)
**8**: Obtain the parameters Θ={γ,λ} of important variables of target individual $k^{*}$ by solving the minimization problem using the gradient‐based algorithm

Recall that for each individual k (k=1,…,K), we have a pair of survival functions for a fixed number of time points {s} ($s=1,\text{…},T_{max}$), which are $S_{ks}^{BB}$ predicted by the BBS model and $S_{ks}^{JM}$ formulated based on the joint model. In this paper, we calculate the distance between $S_{ks}^{BB}$ and $S_{ks}^{JM}$ based on the $L_{2}$‐norm for every individual k:

(8) $$\begin{array}{cc} D(S_{ks}^{BB},S_{ks}^{JM}) & =∥S_{ks}^{BB}-S_{ks}^{JM}{∥}_{2}^{2}\\ & =\sum_{s=1}^{T_{max}}{\left(S_{ks}^{BB}-S_{ks}^{JM}\right)}^{2}. \end{array}$$

Hence, the optimal parameters Θ={γ,λ} in $S_{ks}^{JM}$ can be obtained by minimizing the weighted distance between $S_{ks}^{BB}$ and $S_{ks}^{JM}$ for all K individuals:

(9) $$\min_{\Theta }\sum_{k=1}^{K}w_{k}\cdot \sum_{s=1}^{T_{max}}{\left(S_{ks}^{BB}-S_{ks}^{JM}\right)}^{2}$$

substituting $S_{ks}^{JM}$ with Equation (7), we finally obtain an unconstrained nonlinear minimization problem that can be solved by gradient‐based algorithms:

(10) $$\min_{\Theta }\sum_{k=1}^{K}w_{k}\cdot \sum_{s=1}^{T_{max}}{\left(S_{ks}^{BB}-\prod_{t=1}^{s}\left(1-{logit}^{-1}\left(a_{t}^{0}+\gamma^{⊤}u_{k}+\lambda m_{kt}\right)\right)\right)}^{2}.$$

We write the whole procedure for obtaining the optimal parameters Θ={γ,λ} for the target individual $k^{*}$ using JointLIME in Algorithm 1.

Recall that our goal is to generate a local explanation of the prediction made by the BBS model for a target individual $k^{*}$. Therefore, the weight $w_{k}$ to each individual k in the neighborhood data set is applied to capture the locality. In this paper, we use a Gaussian kernel (Pachón‐García et al., 2024; Ribeiro et al., 2016) to calculate the weight $w_{k}$:

(11) $$w_{k}=\exp\left(-D{\left(k^{*},k\right)}^{2}/2\rho^{2}\right),$$

where $D(k^{*},k)$ is the distance function measuring the distance between each neighborhood individual k and the target individual $k^{*}$ and ρ is the bandwidth, a tuning parameter affecting the neighborhood width. In this paper, following the Normal reference rule (Pachón‐García et al., 2024), we fix ρ as $\rho ={\left\{4/\left[K\left(p+2\right)\right]\right\}}^{1/(p+4)}$, where p represents the number of variables. We use the $L_{2}$ distance as the distance function. The weight $w_{k}$ ensures that the survival functions' distance of those individuals closer to the target individual $k^{*}$ will have a greater impact on finding the optimal values of Θ, which guarantees the local faithful of the interpretation for the target individual $k^{*}$.

After obtaining the optimal parameters Θ={γ,λ}, we further substitute them into the distance function (Equation 8) and then calculate the distance $D^{*}$ between the survival functions generated by the BBS model ($S_{k^{*}s}^{BB}$) and JointLIME ($S_{k^{*}s}^{JM}$), respectively, for the target $k^{*}$:

(12) $$\begin{array}{cc} D^{*}\left(S_{k^{*}s}^{BB},S_{k^{*}s}^{JM}\right) & =∥S_{k^{*}s}^{BB}-S_{k^{*}s}^{JM}{∥}_{2}^{2}\\ & =\sum_{s=1}^{T_{max}}{\left(S_{k^{*}s}^{BB}-S_{k^{*}s}^{JM}\right)}^{2}. \end{array}$$

Since $D^{*}\geq 0$, a smaller $D^{*}$ indicates that JointLIME performs better in approximating the BBS model. We use $D^{*}$ to evaluate the quality of the approximation of JointLIME to the BBS model.

## 7 APPLICATION: CREDIT RISK ANALYSIS FOR US MORTGAGES

### 7.1 Data

**FIGURE 2:**
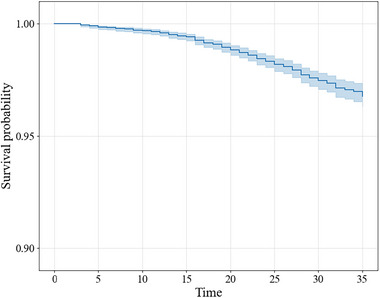
Kaplan–Meier estimate of survival function.

We use the Single Family Loan‐Level Data Set publicly available from Freddie Mac^5^ to illustrate JointLIME in the credit risk analysis. Freddie Mac provides a sample data set containing monthly performance information for 50,000 loans which are randomly selected each vintage year, starting from 1999.^6^ Considering computational capabilities, we use 10,399 loans with 285,462 observations issued from October 1999 to December 1999 and follow their performance for 35 months ($T_{max}=35$). We use the definition of default that corresponds to the time when the borrower is 90 days or more past due, and approximately 2.3% of the loans experienced default during the analysis period. Figure 2 shows the Kaplan–Meier estimate of the survival function over time.

The details of the selected time‐invariant covariates to be used in this paper (Hu & Zhou, 2019; Medina‐Olivares et al., 2022; Wang et al., 2020) are shown in Table 2. Note that we standardize the numerical covariates in JointLIME.

**TABLE 2:** Description of time‐invariant covariates.

Covariate	Type	Description	Mean	Median	SD
FICO score	Numerical	Credit score of the borrower	710.70	716.00	52.50
CLTV	Numerical	Original combined loan‐to‐value ratio	78.05	80.00	15.42
Loan balance	Numerical	Original unpaid principal balance ($1000)	122.35	115.00	53.49
DTI	Numerical	Original debt‐to‐income ratio	33.79	34.00	10.50
One borrower	Categorical	Either one borrower (= 0) or more (= 1)	0.62	1.00	0.49
Loan purpose	Categorical	Whether the loan purpose is refinance (= 0) or purchase (= 1)	0.74	1.00	0.44

For the endogenous TVC, following Medina‐Olivares et al. (2022), we use the difference between the implicit interest rate ($r_{ij}$) and the original fixed interest rate ($R_{i}$), as this provides a balanced reflection of both scheduled and observed loan information. We first calculate the scheduled instalment amount ($A_{ij}$) using the available original loan amount ($P_{i0}$), the fixed interest rate, and the loan term in the data set. The implicit rate ($r_{ij}$) is then calculated based on the observed unpaid principal balance ($P_{ij}$), the scheduled instalment amount ($A_{ij}$), and the available original loan amount ($P_{i0}$) over the remaining mortgage period, as per Equation (13).

(13) $$P_{ij}=P_{i0}{\left(1+r_{ij}\right)}^{j}-A_{ij}\frac{{\left(1+r_{ij}\right)}^{j}}{r_{ij}}+\frac{A_{ij}}{r_{ij}},\;j=1,\text{…},t_{i}.$$

The observed endogenous TVC is represented by $y_{ij}=r_{ij}-R_{i}$. This implies that if repayments adhere to the schedule, the implicit interest rate aligns with the fixed rate set at origination. Conversely, any deviation in the principal balance, whether an increase or decrease, alters the implicit interest rate accordingly. It should be noted that the proposed endogenous TVC fluctuates due to the data provider's practice of rounding the current unpaid principal balance to the nearest $1000 for the first 6 months of each loan. This rounding is also reflected in the implicit interest rate, depending on whether the rounded value is higher or lower than the scheduled amount.

### 7.2 Results

**FIGURE 3:**
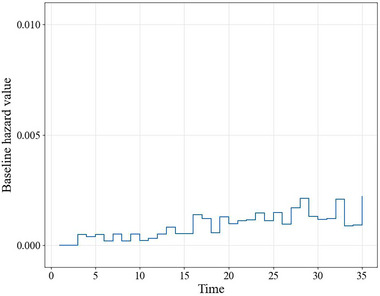
Nelson–Aalen estimate of baseline hazard.

To illustrate the explanations generated by JointLIME, we first randomly select 10 defaulted mortgages and 10 censored mortgages as target individuals. And the rest of the 10,379 mortgages are used as the training set. As mentioned in Section 5.2, the baseline hazard is fitted based on the training set using the Nelson–Aalen estimator (Figure 3). It can be seen that since the data set is extremely imbalanced as mentioned in Section 7.1, the baseline hazard values based on the training set are overall very small but show a slight upward trend over time.

We present the results of the longitudinal model in Section 7.2.1. Section 7.2.2 shows the explanations produced by JointLIME for the target individuals and we also compare them with the explanations produced by SurvLIME.

Experiments were conducted on an Intel Core i9‐13900F CPU at 2.0 GHz with 24 cores and 32 GB of RAM. Generating explanations with JointLIME in this application case took around 40 s per target individual. Note that this time does not include training the Dynamic‐DeepHit or fitting the longitudinal submodel, as once these models are trained and fitted on the training set, they can be reused directly to generate explanations for any individual. Related codes for the experiments can be found on Github: https://github.com/yujiachen02/JointLIME.

#### 7.2.1 Longitudinal model results

We fit the longitudinal model based on the training set using a spline‐based approach. As described in Section 5.1, we fix the positions of spline interior knots at the quantiles of time and determine the number of knots based on the AIC and BIC. Table 3 reports the corresponding AIC and BIC values calculated for nine longitudinal models fitted with one to eight interior knots, respectively. A lower AIC or BIC value means a better fit of the model. Here, longitudinal model with seven interior knots has the lowest AIC and BIC values and therefore is used to calculate the true and unobserved endogenous TVC ($m_{ks}$) for the new individuals. Details of the estimated parameters of the longitudinal model are reported in Table A.1.

**TABLE 3:** Akaike information criterion (AIC) and Bayesian information criterion (BIC) values for longitudinal models.

	Number of interior knots							
	1	2	3	4	5	6	7	8
**AIC**	731,728	707,164	652,769	613,514	567,951	504,160	**495,836**	500,034
**BIC**	731,844	707,301	652,927	613,694	568,152	504,382	**496,079**	500,298

*Note*: The Bold face value means the lowest AIC or BIC value, representing a better fit of the model.

**TABLE 4:** $D^{*}$ of best, mean, and worst approximations for JointLIME and SurvLIME.

	Best	Mean	Worst
JointLIME	0.0003	0.05	1.25
SurvLIME	0.007	0.16	1.28

**FIGURE 4:**
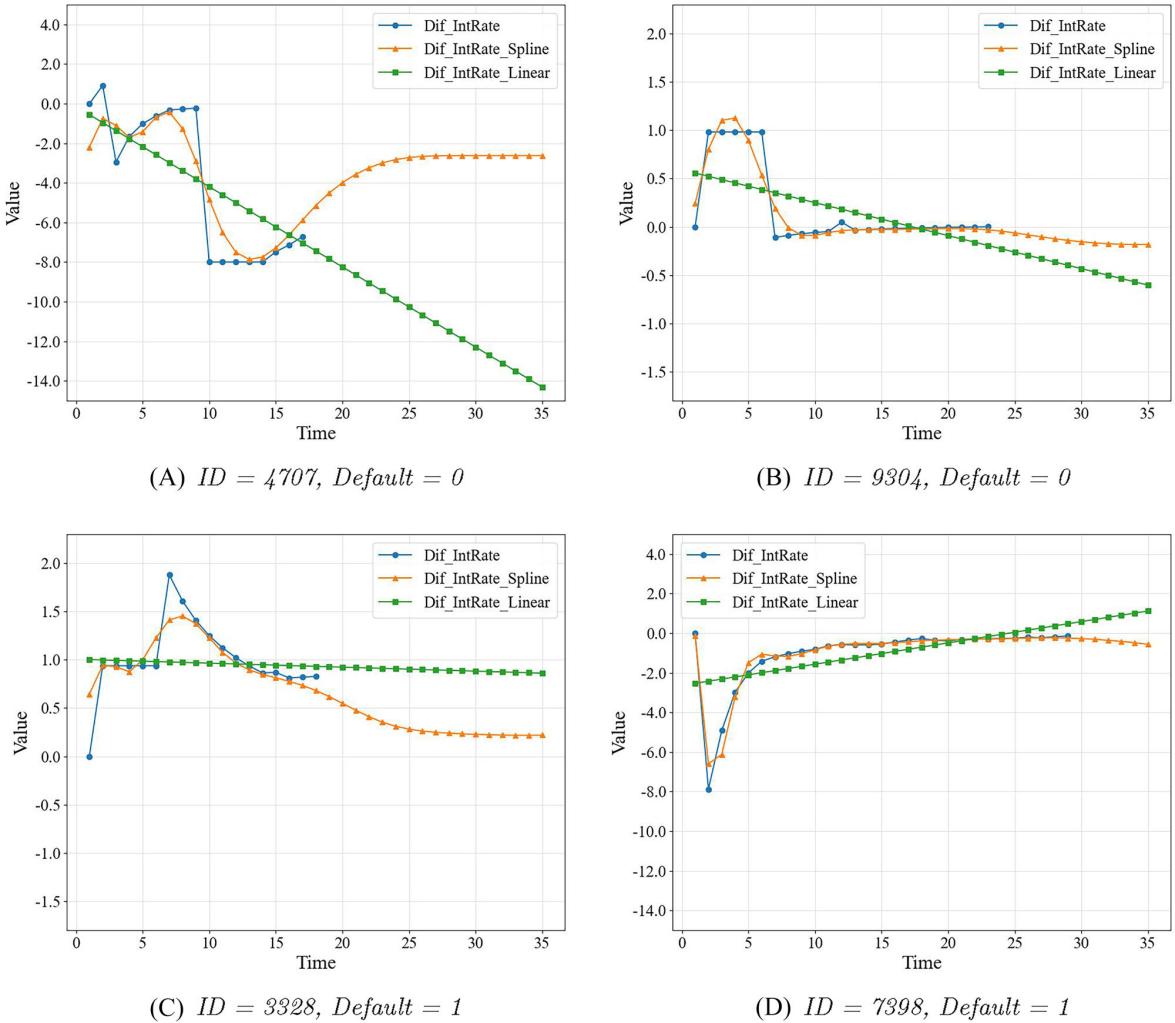
Examples of observed endogenous time‐varying covariate (TVC, in blue), estimated true and unobserved endogenous TVC based on cubic B‐Spline model (in orange), and those estimated based on linear mixed‐effects model (in green).

Figure 4 shows four examples of the estimated $m_{ks}$ based on the cubic B‐spline model (lines in orange), comparing with the observed endogenous TVC (lines in blue), and the estimated $m_{ks}$ based on the linear mixed‐effect model (lines in green).^7^ Panels A and B in Figure 4 show the censored endogenous TVC for two target individuals, and Panels C and D show the endogenous TVC for two target individuals that are defaulted during the analysis period. It can be observed that the spline‐based approach used in this paper demonstrates superior performance in fitting the existing endogenous TVC and offers more reliable predictions for the future trends of the endogenous TVC.

#### 7.2.2 JointLIME versus SurvLIME

**FIGURE 5:**
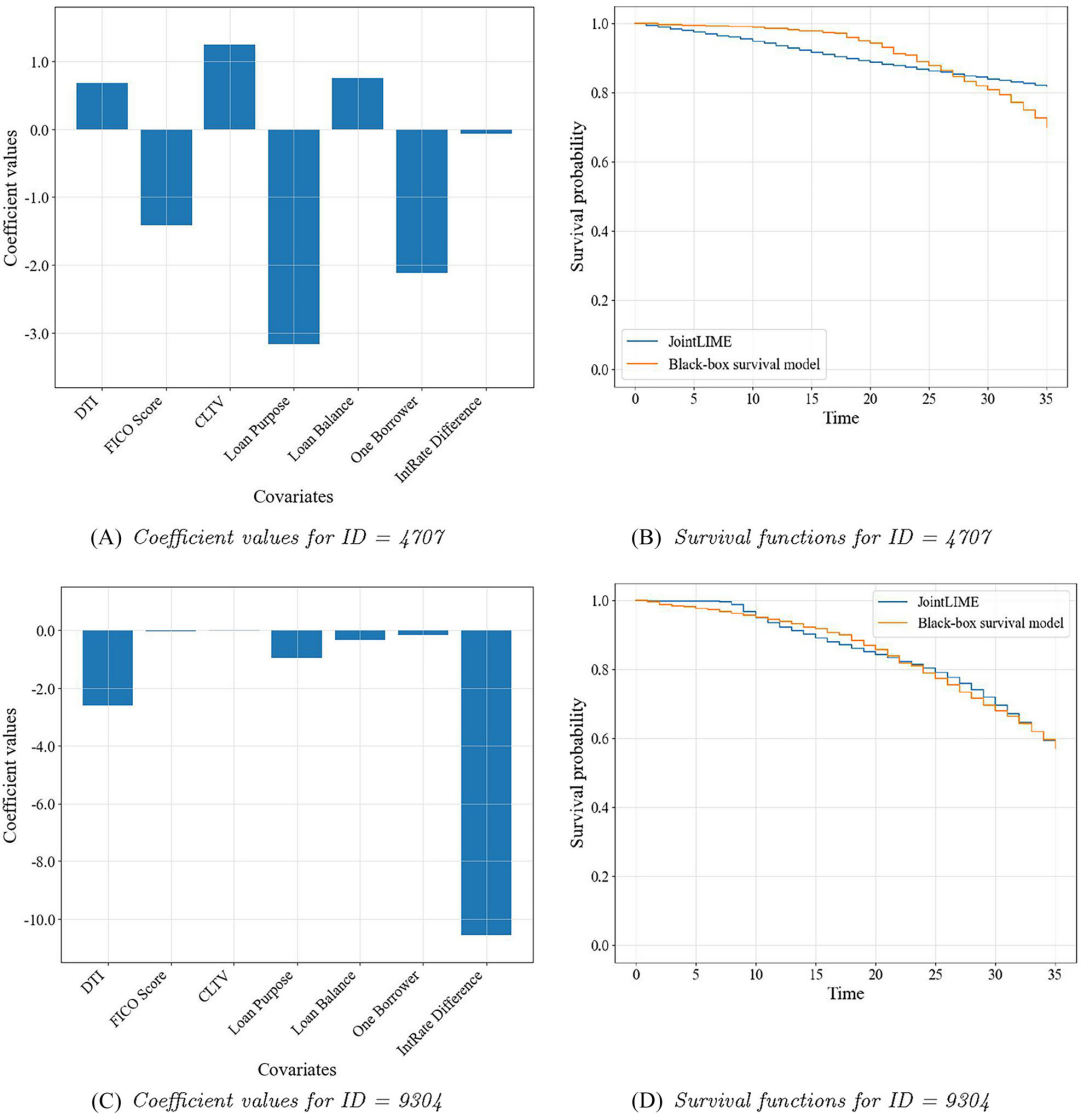
Important variables (left) and survival functions (right) for censored borrowers. CLTV, combined loan‐to‐value ratio; DTI, debt‐to‐income ratio.

**FIGURE 6:**
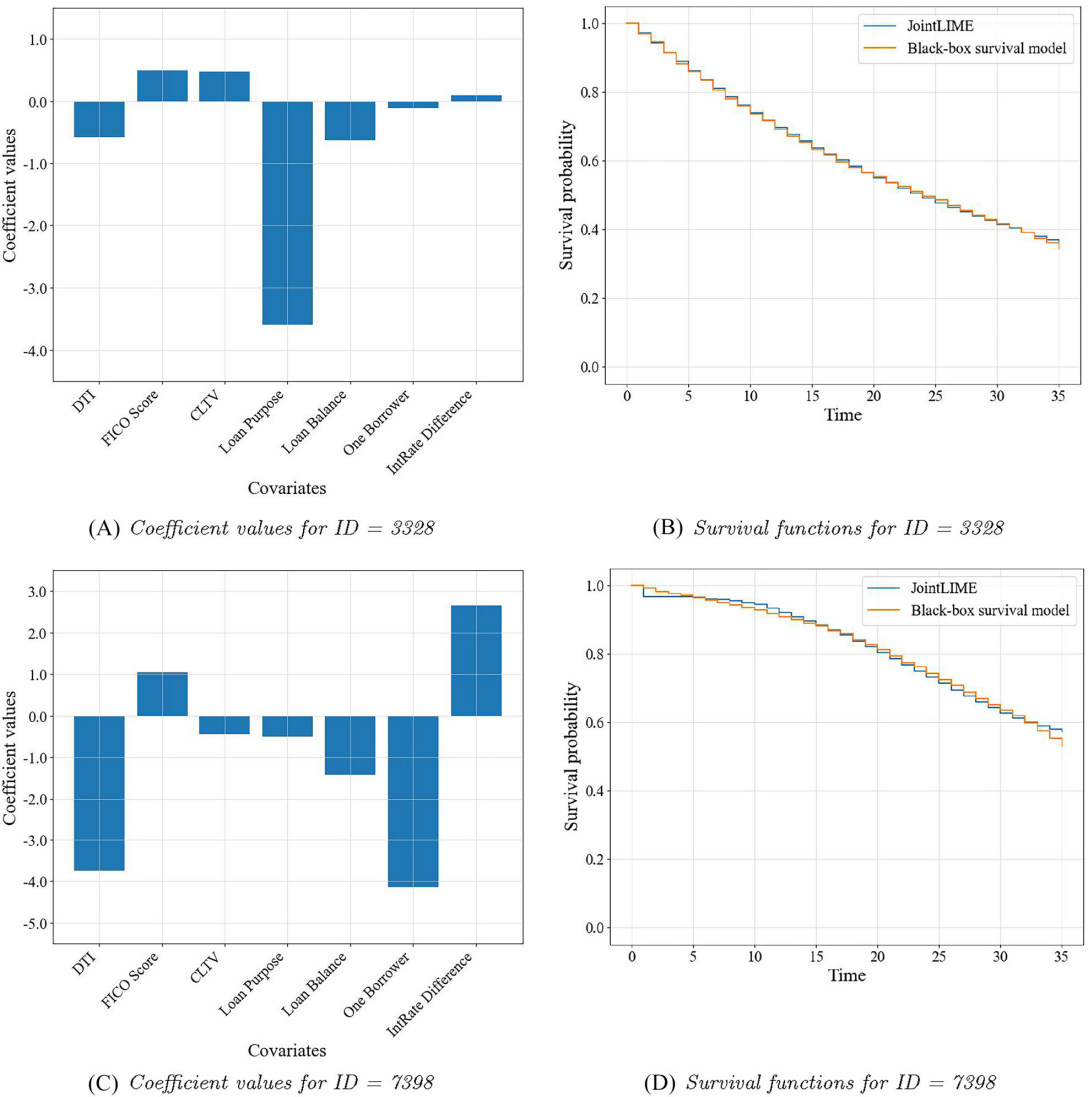
Important variables (left) and survival functions (right) for defaulted borrowers. CLTV, combined loan‐to‐value ratio; DTI, debt‐to‐income ratio.

In this section, we first show the interpretation results generated by JointLIME for the four target individuals whose endogenous TVC have been illustrated in Figure 4. Specifically, Figure 5 illustrates the interpretation results for the two censored borrowers and Figure 6 for the two defaulted borrowers. In both figures, the left bar charts show the optimal parameters generated by JointLIME, which are exactly the coefficients for all seven covariates, with the first six being time‐invariant covariates and the last (IntRate Difference) being the TVC. The absolute coefficient values can then be regarded as the variable importance values. The right line charts illustrate the survival functions (in orange) predicted by the BBS model (Dynamic‐DeepHit), and the survival functions (in blue) approximated by the joint model in JointLIME. The $D^{*}$ values for the target individuals with IDs 4707, 9304, 3328, and 7398 are 0.07, 0.01, 0.002, and 0.006, respectively. Although the values vary, they are all close to 0, indicating strong approximation performance by JointLIME.

It can be seen that for the target individual with ID=4707 (Figure 5B), the survival function approximated by JointLIME is not very consistent with the survival function predicted by the BBS model. Observing its corresponding endogenous TVC (Figure 4A), it is known that the endogenous TVC fluctuates significantly and the absolute values are large. But the predicted survival function remains almost at 1 for nearly half of the time and then declines gently. JointLIME can only approximate the predicted survival model as closely as possible by reducing the impact of the endogenous TVC. Hence, we can see that the coefficient value corresponding to the endogenous TVC (IntRate Difference in Figure 5A) is close to 0. This also leads to the final approximated survival function being nearly linear.

On the contrary, the endogenous TVC for the target individual with ID=9304 (Figure 4B) is relatively stable and has smaller absolute values. The survival function predicted by the “black box” survival model shows a declining trend (Figure 5D). JointLIME therefore increases its influence on the approximated survival function by assigning larger absolute coefficient values to the endogenous TVC (IntRate Difference in 5C). Target individual with ID=7398 in Figure 6 also shows a similar interpretation pattern to the one mentioned above with other time‐invariant covariates also playing relatively important roles.

For the target individual with ID=3328 (Figure 6A,B), the survival function predicted by the BBS model shows a close‐to‐linear pattern. Therefore, the TVC has a lesser impact, while the importance of other time‐invariant covariates is increased.

Similar interpretation results can be found for other target individuals. Overall, the survival function predicted by the BBS model and the approximated survival function obtained by JointLIME is very close to each other for almost all the target individuals. Moreover, JointLIME can consider the impact of the TVC on the output of the BBS model and provide reasonable explanations by assigning importance values to covariates.

In this paper, we also use SurvLIME to produce the explanation results for the target individuals. Since SurvLIME cannot handle the endogenous TVC, we treat the endogenous TVC at each time point as time‐independent covariates. Specifically, there are 35 time points, therefore, in addition to the original six time‐invariant covariates, 35 more covariates are added. When generating the neighborhood data, we follow the method in JointLIME of randomly selecting the endogenous TVC from the training set, then we convert them into 35 time‐independent covariates. Missing values in the added 35 covariates are replaced by the mean value of the endogenous TVC at that time point in the training set. We follow Kovalev et al. (2020) and Pachón‐García et al. (2024) for all other settings to run SurvLIME.

**FIGURE 7:**
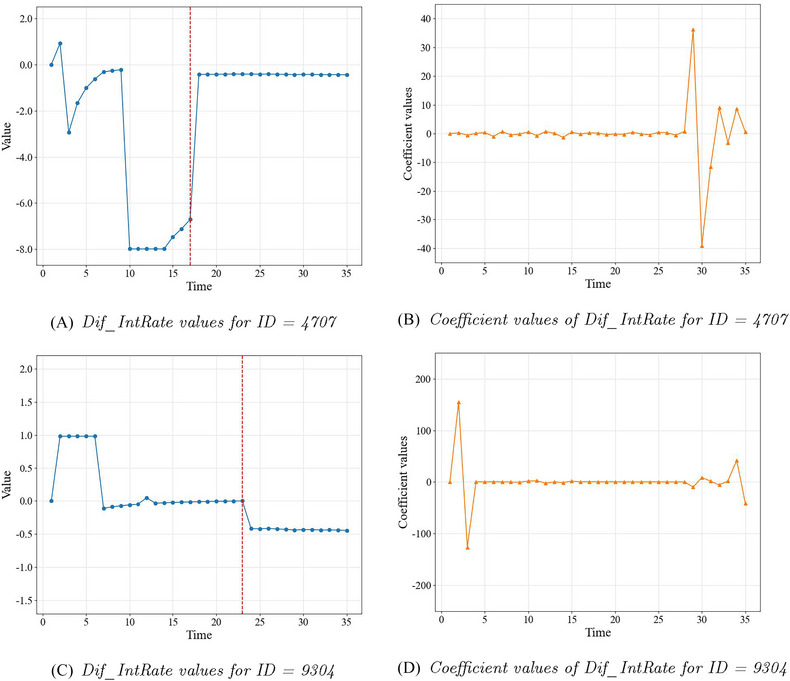
Dif_IntRate in SurvLIME: values of 35 variables (left) and corresponding coefficient values (right) for censored borrowers.

**FIGURE 8:**
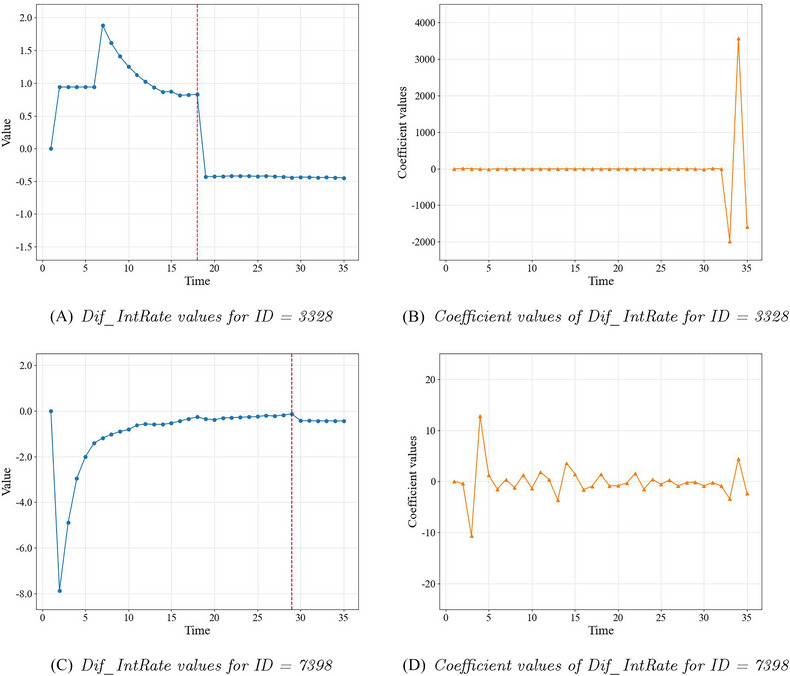
Dif_IntRate in SurvLIME: values of 35 variables (left) and corresponding coefficient values (right) for defaulted borrowers.

Here we focus on the coefficient values produced by SurvLIME for the 35 added covariates. Left line charts in Figures 7 and 8 show the values of the endogenous TVC. The red dashed line represents the endpoint of the observed endogenous TVC and the values after the endpoint are the fitted mean values as described above. Note that the value of the endogenous TVC at each time point represents the value of a corresponding covariate. The coefficient values of the corresponding 35 covariates are illustrated in the right line charts in Figures 7 and 8 for the four target individuals. For example, the value for “Dif_IntRate_1” in Panel B represents the coefficient value of the covariate containing values of the endogenous TVC at time point = 1. It can be seen that the coefficients generated by SurvLIME do not have reasonable connections with the corresponding values of the covariates and therefore cannot be used as reliable explanations.

**FIGURE B.1:**
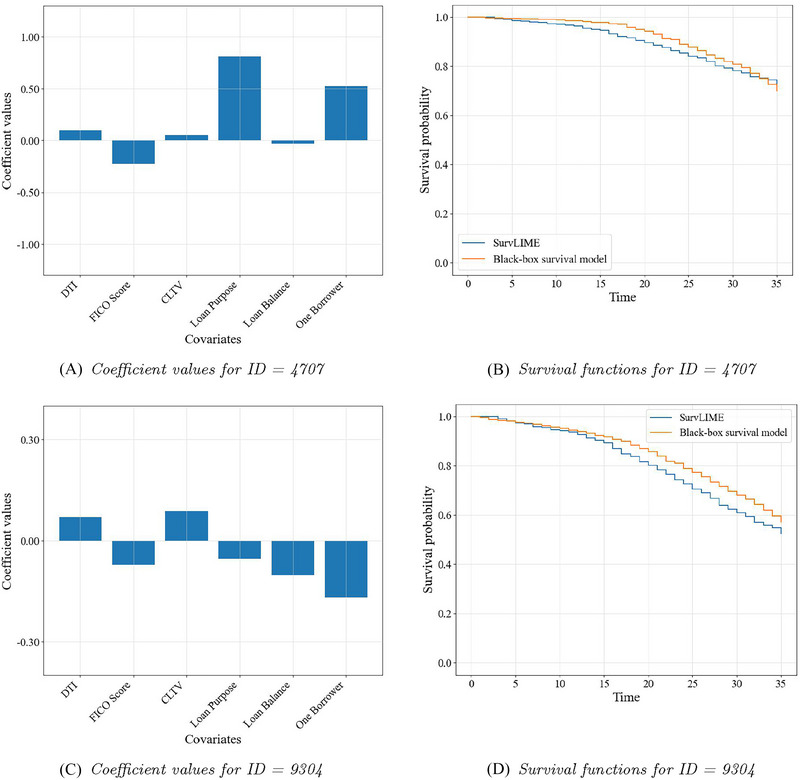
SurvLIME: Time‐invariant covariates (left) and survival functions (right) for censored borrowers. CLTV, combined loan‐to‐value ratio; DTI, debt‐to‐income ratio.

The coefficient values of the other six time‐invariant covariates and the approximation of the survival functions generated by SurvLIME are reported in Appendix B. As shown in Figure B.1 and B.2, SurvLIME generates worse approximations of survival functions compared to JointLIME for most of the target individuals.

**FIGURE 9:**
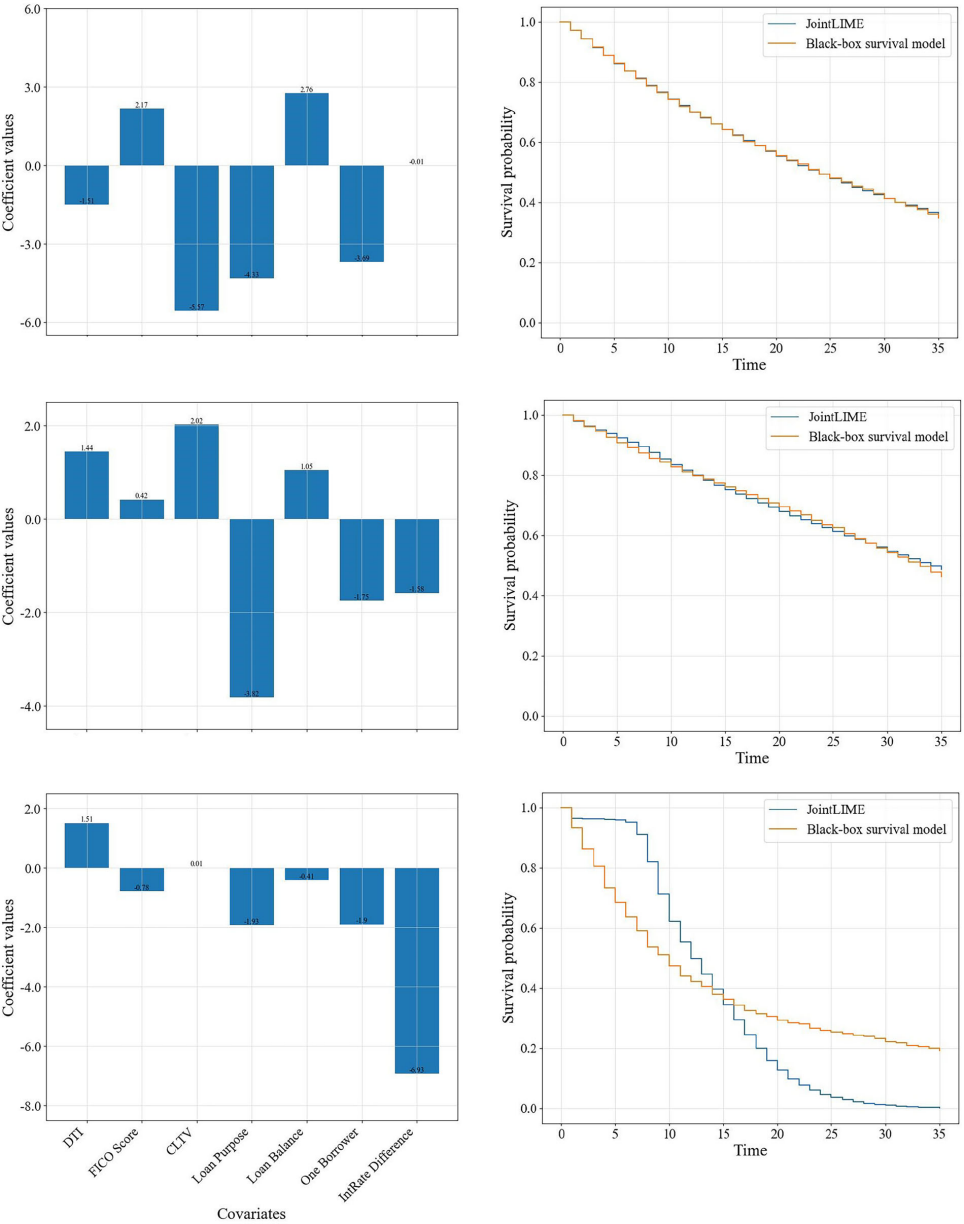
The best, mean, and worst approximations for JointLIME. CLTV, combined loan‐to‐value ratio; DTI, debt‐to‐income ratio.

**FIGURE B.3:**
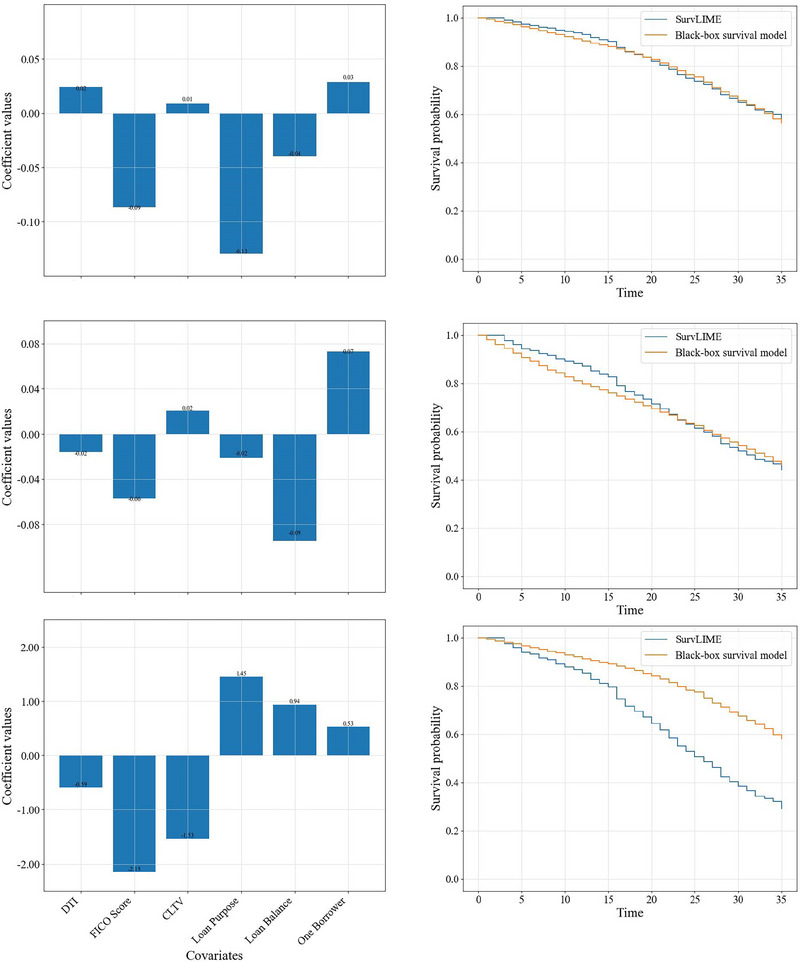
The best, mean, and worst approximations for SurvLIME. CLTV, combined loan‐to‐value ratio; DTI, debt‐to‐income ratio.

To further demonstrate the performance of JointLIME, we randomly select a larger group of target individuals that includes 100 loans (30 defaults and 70 nondefaults^8^) and obtain the explanations generated by JointLIME. We report in Figure 9 the best (plots in the first row), mean (plots in the second row), and worst (plots in the third row) approximations in accordance with the distance $D^{*}$ calculated for these 100 target individuals. The left plots show the importance values of variables. The right plots show survival functions obtained from the BBS model (Dynamic‐DeepHit) and from the joint model approximation. It shows that although in the worst case, the survival function generated by JointLIME does not perfectly match that of the BBS model, the overall approximation provided by JointLIME remains reliable. We also obtain explanations for these 100 target individuals using SurvLIME, and the results are reported in Figures B.3 and B.4. Table 4 presents the $D^{*}$ values for JointLIME and SurvLIME under the best, mean, and worst approximation cases. It can be seen that JointLIME has a better approximation performance than SurvLIME in all three cases.

## 8 CONCLUSIONS AND FUTURE RESEARCH

This paper presents JointLIME, a new interpretation method designed to explain the BBS models, specifically those equipped to manage endogenous TVCs. The main idea of JointLIME is to apply the joint model to approximate the survival functions predicted by the BBS models in a local area around a new individual. The coefficients of covariates in the joint model can then be regarded as the quantitative impacts on the survival predictions. By applying JointLIME to real‐world mortgage data, it is evident that within a certain prediction period, JointLIME is capable of assessing the influence of the variation in endogenous TVCs. It also assesses the effects of static values of time‐invariant covariates. This process results in generating reasonable importance values for each covariate, showing how they affect predicted survival probabilities.

To the best of our knowledge, JointLIME is the first interpretation method in survival analysis that considers endogenous TVCs. It has been shown in Section 7.2.2 that SurvLIME, being incapable of including endogenous TVCs, cannot produce logical explanations. Moreover, we employ a spline‐based approach to fit the endogenous TVC and further use a Monte Carlo method to generate true and unobserved endogenous TVC for a new individual within a specified prediction period. These estimates enable JointLIME to effectively explain the survival predictions. In contrast, replacing the Cox model in SurvLIME with an extended Cox model allows for incorporating TVCs. However, SurvLIME lacks an endogenous TVC modeling phase, unlike the joint model. Because of this, SurvLIME cannot create complete estimates of endogenous TVC for a new individual over a specific prediction period. Consequently, it fails to provide effective explanations. In addition, JointLIME is the first interpretation method to consider the discrete survival time. By converting the discrete survival model into the standard continuous survival model typically employed in the joint model, JointLIME can also be easily adapted for cases assuming the continuous survival time.

Overall, JointLIME is a powerful tool for interpreting BBS models that incorporate endogenous TVCs by providing importance values for various covariates. It is essential to understand that JointLIME is designed to include endogenous TVCs, which are typically behavioral variables. Thus, the BBS models it explains are better suited for behavioral scoring predictions rather than for determining credit scores at the time of loan application. This means that JointLIME is not intended to meet the stringent interpretability standards often mandated by regulations for explaining loan application rejections. Instead, its appropriate use case is within a bank's internal processes, where the focus is on deriving behavioral scores based on customer behavior to predict and enhance future profits or potential purchases. By employing BBS models, banks can achieve more accurate predictions, and JointLIME aids in improving the interpretability of these models. In these scenarios, the required level of interpretability is relatively lower. The explanations provided by JointLIME help professionals better understand and evaluate the validity and feasibility of the predictions generated by BBS models. At the same time, it is important to note that all proposed interpretation methods, including JointLIME, are based on certain assumptions (e.g., linear approximation for explanations). Moreover, given their complexity, careful consideration is required regarding the adoption and implementation of these methods. While they can enhance users' understanding and reshape their cognitive processes, they can also carry risks, such as reinforcing biases and leading to suboptimal or biased decisions (Bauer et al., 2023). How to assess and measure the reliability of interpretation methods and their impact on decision making is beyond the scope of this paper, but is worth for further exploration.

It should be noted that the applicability of JointLIME extends beyond credit risk modeling and can be effectively employed in various fields. For example, in medical research, survival analysis is of critical importance, and endogenous TVCs like blood pressure and other vital signs often serve as key variables.

In this paper, we only consider a static importance value for each covariate in JointLIME. It would be interesting to extend the existing explanation framework to generate time‐varying coefficients of each covariate, thereby further investigating how the changing values of endogenous TVCs over time impact the predictions. Another direction for future research would be to consider other distance metrics when measuring the distance between the two survival functions derived from the BBS model and the joint model, respectively. Moreover, when constructing the neighborhood data set, we sample the endogenous TVC from the training set, which is feasible when the training set is large enough. However, when the available data are extremely limited, we need to consider alternative methods of sampling endogenous TVC. One such approach could involve the use of generative adversarial networks (Dash et al., 2019; Yale et al., 2020) to create synthetic data of the endogenous TVC. This is also a direction for further research.

## Figures and Tables

**FIGURE B.2:**
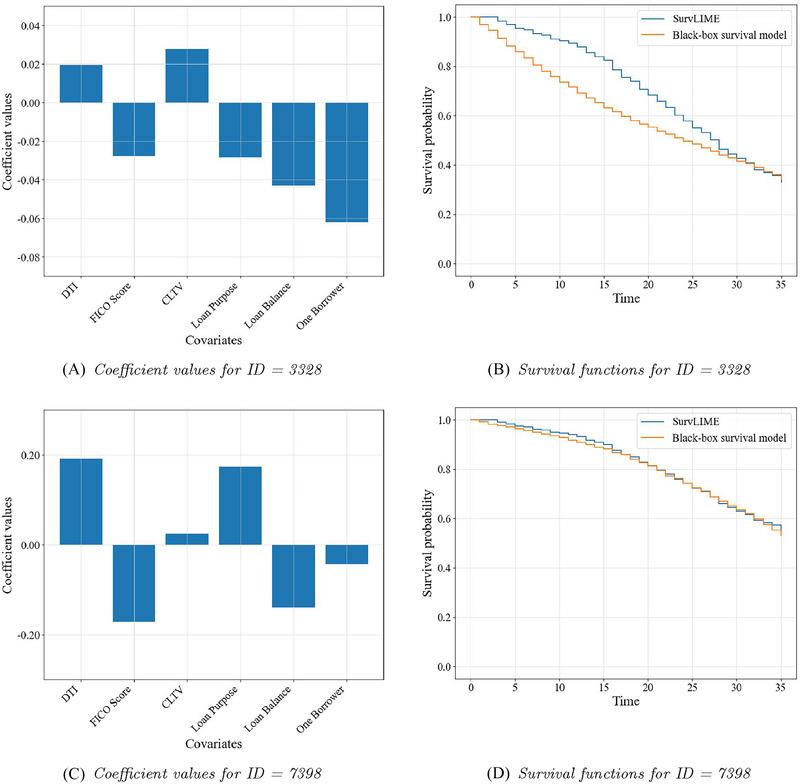
SurvLIME: Time‐invariant covariates (left) and survival functions (right) for defaulted borrowers. CLTV, combined loan‐to‐value ratio; DTI, debt‐to‐income ratio.

**FIGURE B.4:**
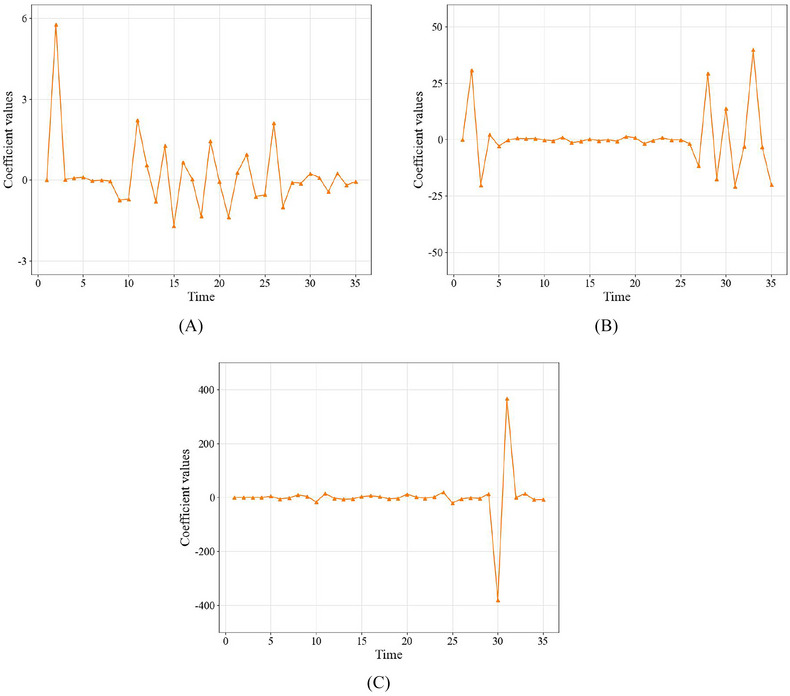
Coefficient values for Dif_IntRate in SurvLIME: best (A), mean (B), and worst (C) approximations of SurvLIME.

## Footnotes

1
$T_{max}$ represents the maximum time for observed endogenous TVCs. Please note that we use s to distinguish it from j.

2 It is important to emphasize that the goal of this paper is to propose an interpretation method for BBS models with endogenous TVCs, but not to optimize the predictive performance of these models. Therefore, rather than exploring a larger number of combinations or using a grid search method, we randomly select 50 combinations, prioritizing computational efficiency and leveraging insights from existing literature. For example, in Blumenstock et al. (2022), DeepHit, a model similar to Dynamic‐DeepHit but without a temporal attention mechanism, was applied to credit risk modeling, where seven hyperparameters were tuned using 30 combinations.

3 The discriminative performance is evaluated using the time‐dependent concordance index, which is provided by Dynamic‐DeepHit (Lee et al., 2019).

4
$k^{*}$ is included in the K individuals.

5
https://www.freddiemac.com/research/datasets/sf‐loanlevel‐dataset.

6 Freddie Mac data sets from more recent years, particularly those following the global financial crisis, exhibit very few default events, often with default rates falling below 1%. This results in an extremely imbalanced data set, which can negatively affect the performance of interpretation methods like LIME and SHAP (Chen et al., 2024). Therefore, using data sets from the early 2000s, where default rates were higher, may be more appropriate. It is important to acknowledge that class imbalance could pose a challenge and requires careful consideration of its effects.

7 Medina‐Olivares et al. (2022):

$$y_{ij}=\underset{m_{ij}}{\underset{︸}{\alpha^{0}+\beta_{i}^{0}+\beta_{i}^{1}\cdot j}}+\varepsilon_{ij},\;j=1,\text{…},t_{i},$$

where $\alpha^{0}$ is the fixed effect (intercept), $\beta_{i}^{0}$ and $\beta_{i}^{1}$ are random effects (intercept and slope, respectively).

8 We select fewer defaults to ensure that the default rate in the training set remains at a more reasonable level (not below 2%), as interpretation methods tend to exhibit unstable performance on extremely imbalanced data sets (Chen et al., 2024).

## APPENDIX A ESTIMATED PARAMETERS OF LONGITUDINAL MODEL

**TABLE A.1:** Estimated parameters of longitudinal model.

Parameters	Mean	SD	Parameters	SD
$\beta_{1}$	−0.9898	0.0468	$b_{1}$	4.6937
$\beta_{2}$	−0.3443	0.0235	$b_{2}$	2.2840
$\beta_{3}$	−0.6035	0.0121	$b_{3}$	1.0123
$\beta_{4}$	−0.3932	0.0097	$b_{4}$	0.7334
$\beta_{5}$	−0.4366	0.0094	$b_{5}$	0.7116
$\beta_{6}$	−0.4008	0.0094	$b_{6}$	0.6460
$\beta_{7}$	−0.4014	0.0104	$b_{7}$	0.7040
$\beta_{8}$	−0.4157	0.0124	$b_{8}$	0.7076
$\beta_{9}$	−0.4286	0.0150	$b_{9}$	0.8489
$\beta_{10}$	−0.4236	0.0121	$b_{10}$	0.6319
			Residual	0.4010
